# Anticancer Effect of Spices Used in Mediterranean Diet: Preventive and Therapeutic Potentials

**DOI:** 10.3389/fnut.2022.905658

**Published:** 2022-06-14

**Authors:** Wamidh H. Talib, Mallak J. AlHur, Sumaiah Al.Naimat, Rawand E. Ahmad, Arkan Hadi Al-Yasari, Anfal Al-Dalaeen, Samar Thiab, Asma Ismail Mahmod

**Affiliations:** ^1^Department of Clinical Pharmacy and Therapeutics, Applied Science Private University, Amman, Jordan; ^2^Office of Scientific Affairs and Research, King Hussein Cancer Center, Amman, Jordan; ^3^Faculty of Biotechnology, Itmo University, St. Petersburg, Russia; ^4^Department of Clinical Nutrition and Dietetics, Faculty of Pharmacy, Applied Science Private University, Amman, Jordan; ^5^Department of Pharmaceutical Chemistry and Pharmacognosy, Applied Science Private University, Amman, Jordan

**Keywords:** spices, cell apoptosis, chemo-prevention, anti-angiogenesis, ginger

## Abstract

Cancer is one of the leading causes of death worldwide, with almost 10 million cancer-related deaths worldwide in 2020, so any investigation to prevent or cure this disease is very important. Spices have been studied widely in several countries to treat different diseases. However, studies that summarize the potential anticancer effect of spices used in Mediterranean diet are very limited. This review highlighted chemo-therapeutic and chemo-preventive effect of ginger, pepper, rosemary, turmeric, black cumin and clove. Moreover, the mechanisms of action for each one of them were figured out such as anti-angiogenesis, antioxidant, altering signaling pathways, induction of cell apoptosis, and cell cycle arrest, for several types of cancer. The most widely used spice in Mediterranean diet is black pepper (*Piper nigrum* L). Ginger and black cumin have the highest anticancer activity by targeting multiple cancer hallmarks. Apoptosis induction is the most common pathway activated by different spices in Mediterranean diet to inhibit cancer. Studies discussed in this review may help researchers to design and test new anticancer diets enriched with selected spices that have high activities.

## Introduction

Since ancient times spices and herbs have been extensively used as a food flavoring and traditional medicines (1). Based on history and several current studies, the Mediterranean region has been recognized across generations with a rich reserve of natural medicinal plants (2). As well, the consumption of the main components of the Mediterranean diet has shown a diverse array of health benefits due to the presence of abundant natural phytochemicals (3). Besides, it is believed that using herbs and spices in the traditional Mediterranean diet is associated with emphasizing its medicinal properties and protecting against chronic diseases, including cancer (3, 4). According to statistical analysis, the Mediterranean area exhibited a lower incidence rate of different types of cancer compared to other areas of the world (5). Several studies have reported the antioxidant, anti-inflammatory, and immunomodulatory impact of spices, which may be correlated with the prevention and treatment of cancer (1). Moreover, polyphenols are the main bioactive chemical compounds found in spices and culinary herbs (6). The recent research demonstrated the role of dietary polyphenols as powerful antioxidant and anticancer agents along with many medicinal properties (7–10). It revealed chemo-preventive potency represented by modulation of different processes and biomarkers, such as tumor cell apoptosis, cell cycle progression, inflammation mediators, cell invasion, and metastasis (11). In literature, there are countless spice-derived secondary metabolites that exhibited potential for cancer prevention, however; they are still under research and development (12, 13). This review summarized some studies about well-known spices in the Mediterranean diet demonstrating their anticancer effects and mechanisms of action. Studies discussed in this review may provide a solid base for researcher and nutritionists to develop effective anticancer nutrition.

## Spices in The Mediterranean Diet: Flavor Characteristics and Traditional Use

Spices are used in different Mediterranean food recipes to impart aroma, color, and taste to food preparations and sometimes mask undesirable odors (14). Spices refer to the dried part of a plant that contains volatile oils or aromatic flavors such as buds (cloves), bark (cinnamon), root (ginger), berries (black pepper), seeds (cumin, coriander) (15, 16). Recently, measurements of dietary intake of spices are gaining much significance as various phytochemicals present in spices, have been recognized to have health-promoting benefits (17). Spices are used in traditional Mediterranean cuisines such as soups, cooked lamb roast, fish preparations, marinades, bouquet garni, baked fish, rice, salads, occasionally with egg preparations, dumplings, vinegar, jams, and marmalades (15).

Spices such as ginger (*Zingiber officinale*), that gives a refreshing pleasant aroma, biting taste, and carminative property, which make it an indispensable food ingredient in most Mediterranean food recipes (16), is used in different forms such as fresh ginger, dry ginger, ginger powder, ginger oil, and ginger paste to enhance both sweet and savory traditional Mediterranean recipes (18).

Rosemary (*Rosmarinus officinalis*), an aromatic herb that has been known from ancient times as a memory herb, a native to the Mediterranean from Spain to the Balkans and into North Africa (14, 19). At present, rosemary is widely cultivated in Spain, Morocco, Tunisia, France, Algeria, Portugal, and China (20). The fresh and dried leaves of rosemary are used frequently in traditional Mediterranean cuisine as they have a bitter astringent taste and are aromatic, dried, and powdered leaves (21). Some spices are used in small amounts because of their intense flavor, such as clove (*Syzygium aromaticum* L.), clove used as a whole or ground form or in oil form that is used in a small amount, for example, curry powder uses 2 % (mild) to 3 % (sweet) by weight of ground clove buds (15). Clove oil is one of the most important essential oils used for flavoring all kinds of food products, such as sausage, baked goods (22).

Black Cumin (*Nigella sativa L.)* is an ancient spice with a mild odor and warm, bitter taste (23). Black cumin is used as a spice in Middle Eastern cuisines. In ancient Egypt, it was used as a preservative in mummification (24). The seeds of black cumin have a pungent bitter taste and aroma and are used as a spice in Middle Eastern cuisines. The dry-roasted nigella seeds flavor curries, vegetables, and pulses. Black cumin is used in food as a flavoring additive in bread and pickles (24, 25).

The most popular and the most widely used spice in Mediterranean food is black pepper (*Piper nigrum L*) (15). Black pepper contributes toward flavor, taste the predominating ones being taste and flavor, and hence pepper is a multifunctional spice (26). Pepper plays an important role in the cuisines of China, South East Asia, Greece, Italy, and France such as meat dishes, fish preparations, soups, and pickles (27). Some spices such as turmeric (*Curcuma longa L*), is used as color agents, it is made into a yellow powder with a bitter, slightly acrid, yet sweet taste. Fresh spice is much preferred than dried spice in Spain, France, Italy, Greece, Turkey (14, 28). In Egypt as early as 3000 BC. cinnamon (*Cinnamomum cassia*) was used in the Testament of the Bible and there indications (29). Cinnamon is used as a flavoring and coloring agent of the foods. However, it gives a sweet sensation of the food that is enhanced because of the synergetic effect between the sweet taste of sugar and the sweet aroma of cinnamon (16). Moreover, cinnamon makes a tan or brown color for food and it is used in many Mediterranean food recipes such as milk, apple pie, and cinnamon buns (30). Table 1 describes the spices classification and general characteristic.

**Table 1:** Description of spices used in the Mediterranean diet along with their classification and characteristic.

**Spice name**	**Classification of spices**	**Edible part(s)**	**Flavor characteristic**	**References**
Ginger (*Zingiber officinale)*	Hot spices	Rhyzome	Flowery flavor and spicy taste, biting taste, and carminative property.	(16)
Black Peppers (*Piper nigrum L)*	Hot spices	Fruits (Seeds)	A colorant, flavoring, and/or as a source of pungency.	(24)
Rosemary (*Rosmarinus officinalis)*	Herbs	Leaf, terminal shoot	A bitter astringent taste and aromatic.	(20)
Tumeric (*Curcuma longa L)*	Aromatic spices	Rhizome	A colorant, flavoring and medium aromatic.	(28)
Black cumin (*Nigella sativa L)*	Aromatic spices	Fruits (Seeds)	A strong aromatic smell and warm, bitter taste.	(23)
Clove (*Syzygium aromaticum)*	Aromatic spices	Buds	A pungent, strong, and sweet with a bitter, astringent flavor	(22)
Cinnamon (*Cinnamomum cassia*)	Aromatic spices	Stem bark	A sweet and aromatic, and less bitter.	(29)

### Mediterranean Plants Used as Food Additives

There is a growing interest in the use food additives from natural sources to improve taste and appearance, preserve flavor and reduce microorganisms' growth. Because the Mediterranean area has high plant species biodiversity, many of its wild plants can be a useful source for natural food additives (31, 32). In the following paragraphs selected examples of such plants are discussed. *Carex distachya* Desf. (Cyperaceae) is an herbaceous plant that is globally distributed in different habitats. It is a steno-mediterranean species and is known with the Italian name “*carice mediterranea*. *Carex* genus is known of the presence of high content of stilbene derivatives (32). Additionally, flavonoids, including resveratrol, flavolignans, lignans and terpenes were also isolated from the *C. distachya*, as well as other unusual metabolites such as feruloyl monoglyceride macrolactones and dibenzoxazepinones. The high contact of polyphenols made this plant a potential source of natural antioxidants for their food protective effect (32, 33).

*Teucrium chamaedrys* L. (Lamiaceae) is a perennial evergreen euri-mediterranean species that is rhizomatous dwarf shrub. *Teucrium* species are rich in essential oils and is the most abundant source of furanic *neo*-clerodane diterpenes. Other phytochemicals present in this plant include phenylethanoid glycosides, iridoid glycosides and phenolic compounds (32). The medicinal use of *Teucrium chamaedrys* is prohibited in some countries due to its liver toxicity, however, alcoholic extracts are still permitted as flavoring agents, because they are fundamental in providing a bitter aromatic taste (32, 34). *Teucrium polium* L. (Lamiaceae) is another plant from *Teucrium* genus that has medicinal properties and is used as a natural food preservative due to its antioxidant and antimicrobial properties (35, 36). The plant contains phenylethanoid glycosides, *neo*-clerodane diterpenes, iridoid glycosides and flavonoids (32, 37). *Petrorhagia velutina* (Guss.) (Caryophyllaceae) is an annual sud-mediterranean herbaceous plant with a characteristic densely glandular-tomentose stem. Flavonoids C-glycosides were isolated from its leaves, in addition to cinnamoyl glucose esters and phytotoxic chlorophyll derivatives (32). Due to its antioxidant properties, *Petrorhagia velutina* can be used as a natural food preservative, by impeding oxidation, which is a mandatory step in rotting, either by aerobic or anaerobic mechanisms (38). *Arbutus unedo* (Ericaceae) is a steno-mediterranean evergreen small tree that is reported to have various phytochemicals, such as flavonoids, steroids and terpenoids (32). It has antioxidant properties, and thus can also be used as a food preservative (39). *Myrtus communis* (Myrtaceae) is an evergreen small tree that contains important essential oils. Phytochemical investigation of this plant revealed that it contains various monoterpenoids, triterpenes, flavonoids and small amounts of phenolic acids (32). The plant was demonstrated to have antioxidant and antimicrobial properties allowing it to be used as a natural food preservative without altering the nutritional characteristics of the food products (40).

In a study conducted using a number of Mediterranean spices, namely, annatto, cumin, oregano, rosemary, saffron and sweet and hot paprika, to compare the oxidative stability of refined olive oil tested by the Rancimat method with common food additives during storage at different temperatures, reported that the spice extracts have significant stabilizing effects (*P* < 0.05) (41).

## Anticancer Activity of Spices From The Mediterranean Diet: Chemical Constituents and Mechanisms of Action

### Ginger

Ginger (*Zingiber officinale* Roscoe) rhizome is widely used as a spice and folk medicine, affiliated to the Zingiberaceae family, belonging to Southern Asia (42, 43). It has various constituents which may vary as a reason of environmental factors, the place of origin and whether the rhizomes are fresh or dry. Its characteristic odor is due to the presence of volatile oil containing various monoterpenoids and sesquiterpenoids (44). The fresh rhizomes pungency is due to its gingerols content where most abundant one is 6-gingerol (1-[40-hydroxy30-methoxyphenyl]-5- hydroxy-3-decanoate). On the other hand, the pungency of dry rhizomes is due to the shogaols content, such as 6-shogaol, which are formed as a result of thermal degradation of gingerols (44). Additionally, ginger also contains terpenoids, alkanes, paradols and diarylheptanoids (45). The phenolic compounds of ginger including gingerols shogaols and paradols were found to exhibit antioxidant, anti-tumor and anti-inflammatory properties (43, 46, 47).

**Figure 1:**
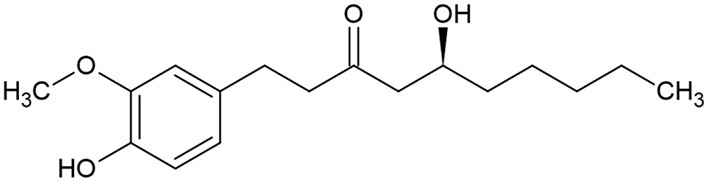
6-gingerol chemical structure.

6-gingerol (Figure 1) was identified as the main active medicinal component of ginger (45). It is usually found as yellow oil and can form a low-melting crystalline solid.

Several mechanisms of action for 6-gingerol have been discussed in many studies, including its chemo-preventive and chemo-therapeutic effects.

The activation of mitogen-activated protein kinase (MAPK) signaling pathway has a role as a possible mechanism behind the chemo-preventive and chemo-therapeutic activity of ginger via the induction of cell arrest against several types of cancer as reported in scientific literature as follows:

One of the studies has investigated the mechanism of the cytotoxic effect of 10-gingerol on human colon cancer cells via the activation of MAPK in a dose-dependent manner, this morphological changes lead to apoptosis that also could be obtained by way of increasing DNA in the sub-G1 phase of the cell cycle (48). The additional study stated the anti-proliferation effect of 6-gingerol on human skin keratinocyte cell lines as a consequence of MAPK and AP-1 signaling pathways (49) and on mouse skin tumor cells through the activation of NF-kappa B(NF-κB), p38 MAPK, and cyclooxygenase-2 (COX-2) expression as reported in a published study (50). Interestingly; another study highlighted the suppression of oral cancer cell growth and inhibition of migration by suppressing the AKT/mTOR signaling pathway and inducing AMP-activated protein kinase (AMPK) which in turn leads to cell arrest and apoptosis (51). 6-Gingerol plays a role in fighting gastric cancer cells along with chemotherapy, particularly Cisplatin, by altering phosphatidylinositol-3-Kinase and Protein Kinase B (PI3K/AKT) signaling pathway; consequently, this will induce cell cycle arrest at the G1 phase (52). Also, 6-Gingerol leads to cell cycle arrest at the G2 phase as well, against oral and cervical carcinoma (53).

Moving to renal cells, cell-cycle G1-phase arrest could be obtained upon 6-Gingerol treatment (54). The impressive study emphasized how 6-Gingerol can induce cell arrest at the G1cell cycle phase of osteosarcoma cells, by dint of AMPK signaling activation, therefore growth abolition (55).

Furthermore, 6-Gingerol could fight human pancreatic cancer cells via the suppression and the downregulation of the ERK/NF- κ B/Snail signal transduction pathway as stated in the reference (56). Reactive oxygen species (ROS) has a role as a possible mechanism behind the chemopreventive and chemotherapeutic activity of ginger via the induction of cell arrest against several types of cancer as reported in scientific literature as follows:

One study approved the anti-tumor activity of 6-dehydrogingerdione which is one of the active extracts of ginger against breast cancer cells in humans that causes growth suppression due to the generation of ROS (57).

Interestingly, another study figured out the inhibitory effect of 6-Gingerol against lung cancer in mice via the generation of ROS (58).

Angiogenesis could be defined as the creation of totally new blood vessels from previously existing endothelium, which is a necessary process in tumor formation (59). It's worth mentioning here the anti-angiogenesis effect of 6-Gingerol via the induction of micro-vessel normalization due to the stabilization of p-VEGFR2/VE-cadherin/β-catenin/actin complex (46). Moreover, an Invitro study showed the inhibitory effect of 6-Gingerol in the suppression of endothelial cell tube formation, therefore it prevents the tumor blood supply (60).

6-gingerol has a suppression effect on the renal cell carcinoma metastasis *in vitro* and *in vivo*, this effect was due to the upregulation of yes-associated protein (YAP) ser127 phosphorylation and the downregulation of YAP levels in cell nuclei that is responsible for cancer cell migration (61).

### Peppers

**Figure 2:**
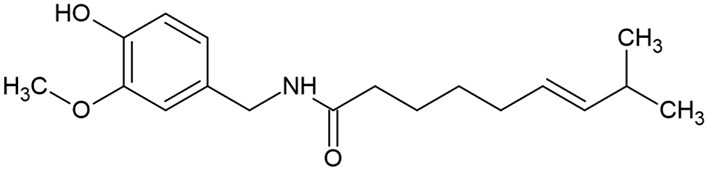
Capsaicin chemical structure.

Capsicum is a genera of pepper, consisting of more than 31 different species including five domesticated species, *C. baccatum, C. annuum, C. pubescen, C. frutescens*, and *C. chinense* (62). Pepper is widely used as a food spice due to its pungency and unique flavor. Pepper contains provitamin A, vitamin E vitamin C, carotenoids and phenolic compounds including capsaicinoids, luteolin, and quercetin (62). Capsicum fruits have been used in the treatment of toothache, infections, coughs, sore throat, rheumatism and for wound healing (62). The main constituent, capsaicin (trans-8-methyl-N-vanillyl-6-nonenamide) (Figure 2), which is an off-white crystalline lipophilic colorless and odorless alkaloid (63), has antioxidant, anti-inflammatory, cytotoxic and antiproliferative effects.

Capsaicin has shown a chemo-therapeutic effect against several types of cancer through the initiation of cancer cell apoptosis (64). Cellular responses upon treatment with capsaicin affect mechanisms of cell death, especially through the downregulation of β-catenin which plays an important role in β-catenin-dependent signaling that is a significant event in the development of malignancies (65). In addition, upregulation of pro-apoptotic genes in other words pro-apoptotic stimuli in tumorigenic cells (66, 67). Furthermore, one study stated the anti-proliferative effect of capsicum through the suppression of FBI-1-Mediated NF-κB Pathway that led to breast cancer cell apoptosis (68).

As discussed previously the anti-angiogenesis effect plays a significant role in killing tumor cells, as it is a possible mechanism of the anti-cancer effect of capsaicin that is figured out *in vivo* and *in vitro* (69). In *in vitro* model, was through the inhibition of tube formation, while *in vivo* through the suppression of vascular endothelial growth factor (VEGF)-induced vessel formation (70, 71).

Capsaicin took part in fighting metastases of cancer, by altering signaling pathways that are important in cell migration (72), Moreover, the anti-invasive effect of capsicin could be done due to the suppression of phosphoinositide 3-kinase (PI3K) signaling cascade and RASrelated c3 botulinum toxin substrate1 (RAC1), that control cancer cell migration (73).

### Rosemary

**Figure 3:**
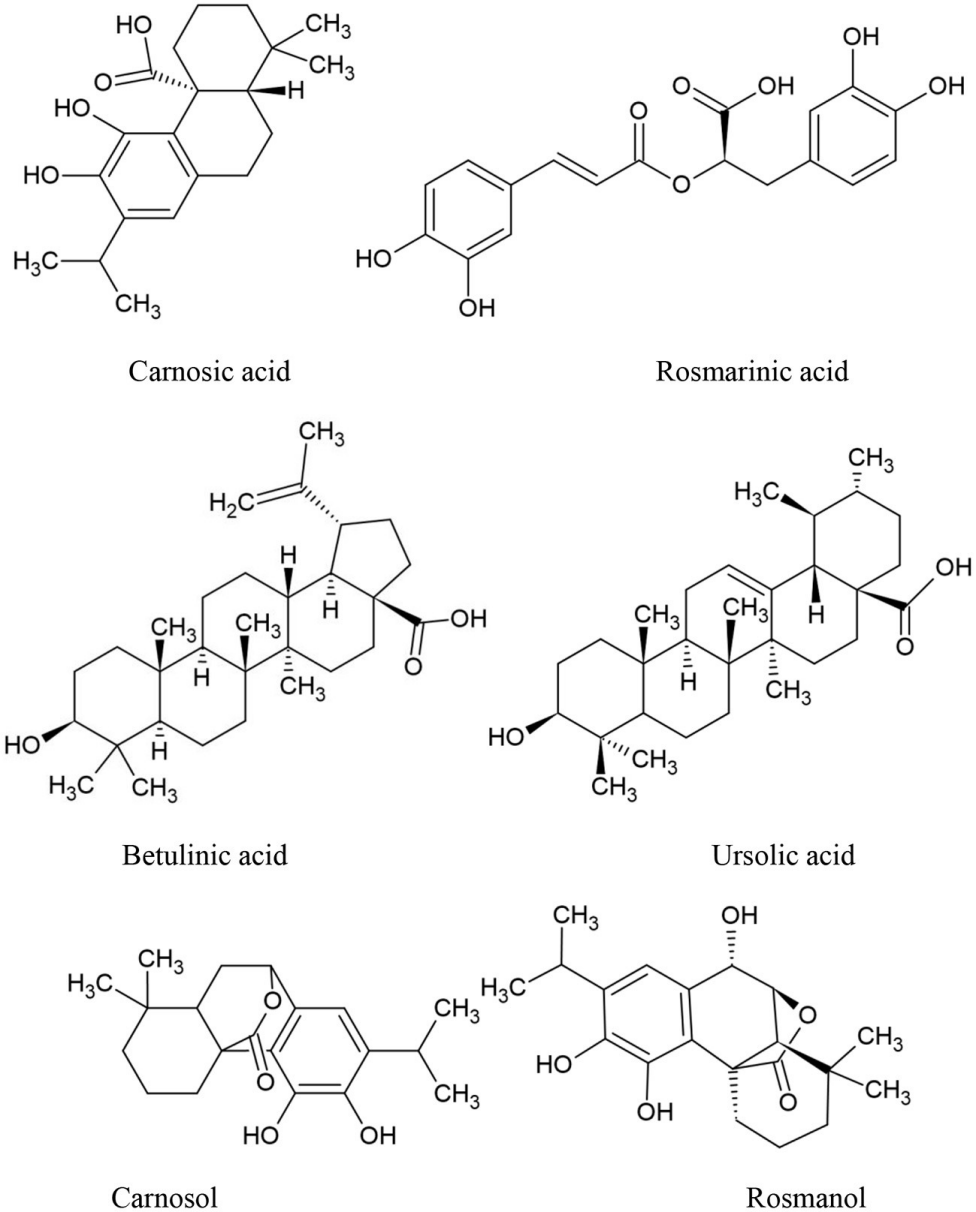
The main components of *Rosmarinus officinalis* chemical structures.

Rosmarinus officinalis L., often known as rosemary, is the scientific name for a Mediterranean plant that is grown in a variety of nations (74). Recently, rosemary extract (RE) was allowed by European Union legislation, allowing food companies to use the label “antioxidant: rosemary extract” on their products (75). Rosemary has been identified as a potential anticancer medication due to its antioxidant properties. It has the ability to act on free radicals and protect DNA, proteins, and lipids from oxidative damage (76), as later discovered, rosemary derivatives are capable of producing cytotoxicity precisely through the generation of ROS in particular conditions. The main active compounds of Rosemary are summarized in Figure 3. Rosemary Extract (RE) has been shown to affect intracellular antioxidant systems by activating the activation of nuclear transcription factor (Nrf) 2 target genes (77) and increasing glutathione levels, with a reduction in its reduced form (GSH) relative to its oxidized form (GSSG) (78).

However, some antioxidants, such as beta-carotene, vitamin E, and vitamin C, have shown mixed results in clinical research addressing their involvement in reducing the risk of cancer formation [the antioxidant impact and anticancer action has been questioned (79–85). Furthermore, Carnosic Acid (CA) and Carnosol (CS) inhibit endothelial cell differentiation, proliferation, migration, and differentiation capacity, as well as other angiogenic capabilities. Several data show that their effects on endothelium and cancer cell development may be related to the programmed cell death stimulation (86).

CA also inhibits cytokine-induced adhesion molecule production and monocyte adherence to endothelial cells via an (NF-kB -dependent mechanism (87, 88).

Histone deacetylases (HDACs), which regulate gene expression by acting on the acetyl group of histones, have abnormal expression patterns that coincide with the beginning of malignancies (89). HDAC2 has been shown to be highly expressed in tumor cells, where it inhibits the production of p53, resulting in a decrease in programmed cell death. The effect of rosmarinic acid (RA) vs. suberoylanilide hydroxamic acid (SAHA), an HDAC inhibitor utilized as an antitumoral medication, on the survival and programmed cell death of tumor cells lines, as well as HDAC production, was recently investigated. Similar to SAHA, RA inhibited cell growth and cancer spheroid formation, as well as causing tumor cell death and blocking HDAC2 expression. RA also decreased cyclins D1 and E1 as well as proliferating cell nuclear antigens, while increasing p21. Finally, a rise in p53 generated from the HDAC2 decrease regulated the protein synthesis of intrinsic mitochondrial apoptotic pathway-related genes (90).

The antineoplastic impact of rosemary could be due to a regulatory effect on the immune system. by enhancing the innate immune response; this enhanced response is attributable to cytotoxic natural killer cells and the formation of an anti-inflammatory cytokine profile, which may aid the immunological response to cancer cells (91). CS inhibited tumor growth, also resulted in a decrease in interleukin-4 (IL-4) and IL-10 (IL-10) and an increase in interferon production (92).

Along with the molecular mechanisms discussed above, additional molecular mechanisms of rosemary have been described and linked to its anticancer effects, including hormone signaling alteration (93), and the ability to interact with a broad range of molecular targets (94, 95). Furthermore, rosemary has recently been shown to boost the expression of genes with known cancer-suppressing capabilities (96). Finally, rosemary phenolic compounds may play a role in a variety of metabolic pathways as well as basic cellular activities and macro-and micronutrient metabolism. These altered pathways may have a clinical impact on the initiation and course of cancer (97, 98). In addition, rosemary extract has been studied in combination with antitumor agents such as 5 Fu, cisplatin, doxorubicin, paclitaxel, tamoxifen, trastuzumab, and Vinblastine. Rosemary extract has a synergistic effect and plays a role in modulating gene expression for enzymes involved in the mechanism of resistance (99–102).

To summarize, while the use of rosemary and its derivatives in the treatment of neoplasms is an interesting topic of research, big and controlled studies are needed to definitively determine the substance's true influence in clinical practice. Taking into account the need to standardize the extraction procedure in order to get REs with consistent antiproliferative properties (103).

### Turmeric

**Figure 4:**
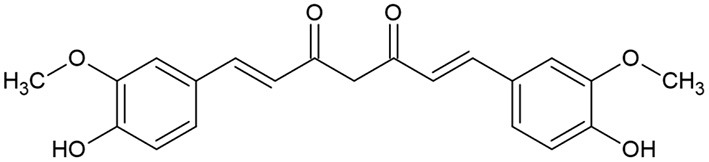
Curcumin chemical structure.

Turmeric (*Curcuma longa*) belongs to Zingiberaceae, which is extensively cultivated for its rhizomes. It is used as spice, preservative and coloring agent in addition to possessing many medicinal applications such as anti-inflammatory, antihyperlipidemic, and antimicrobial activities (104, 105). Turmeric is known to contain poluphenolic compounds known as curcuminoids, including curcumin (Figure 4), demethoxycurcumin and bisdemethoxycurcumin (104, 105).

**Figure 5:**
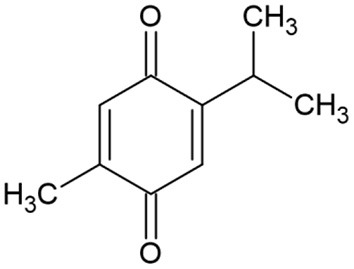
Thymoquinone chemical structure.

Curcumin (Figure 5), the main coloring principal of *Curcuma longa*, is an odorless, yellowish crystalline lipophilic compound, offers a surprising number of health benefits, including anti-inflammatory, antioxidant, chemo-preventive, and chemo-therapeutic characteristics (106, 107).

The intrinsic and extrinsic routes are the two primary pathways that create apoptotic signals. The intrinsic apoptotic pathway works by stimulating the mitochondrial membrane to inhibit anti-apoptotic protein expression (108), curcumin disrupts the mitochondrial membrane potential balance leading to increased suppression of antiapoptotic proteins (109). The extrinsic apoptotic pathway works by increasing death receptors (DRs) on cells and triggering tumor necrosis factor (TNF) related to apoptosis. Curcumin also plays a role in this pathway by increasing the expression of DRs (106, 107, 110).

In addition, findings from *in vitro* and *in vivo* investigations have indicated that curcumin has a powerful cytotoxic effect on many cancer cells by inhibiting oxidative stress and angiogenesis, as well as inducing apoptosis (111).

The PI3K/AKT signaling pathway regulates VEGF expression. Curcumin therapy decreased protein expression levels of PI3K and AKT. Curcumin therapy also dramatically reduced the levels of mRNA expression of VEGF, PI3K, and AKT (112).

Curcumin's anti-inflammatory properties would almost certainly result in its anti-tumor properties, given the close link between inflammation and cancer. Curcumin has been shown to inhibit the development of numerous types of cancer by lowering the production of inflammatory mediators (113).

Increased production of pro-inflammatory molecules such as cytokines, ROS, COX-2, and transcription factors such as NF-B, AKT, activator protein 1 (AP1), and signal transducer and activator of transcription 3 (STAT3) is induced by inflammation, leading to the initiation and progression of cancer. Curcumin's anticancer property comes from its immunomodulatory ability, which it does via interacting with a variety of immunological mediators as it inhibits the transcription of TNF- and, as a result, the expression of inflammatory genes via suppressing NF-B activity. Curcumin's immunomodulatory properties, on the other hand, are directed not only at molecular targets, but also at cellular components like macrophages, dendritic cells, and T and B lymphocytes (114–118).

Curcumin's anti-cancer properties are also related to its interference with the cell cycle and reduction in cyclin-dependent kinases (CDK) expression. CDKs regulate the progression of the cell cycle (119). Curcumin also inhibits STAT3, which is involved in signaling carcinogenic pathways (120).

In the early phases of cancer growth, free radicals and hazardous compounds produced by oxidative stress play a significant role. As a result, substances with antioxidant properties may be useful in avoiding cancer. Curcumin has the ability to trap free radicals, which means it can help prevent cancer from developing. Curcumin prevents DNA damage induced by oxidative causes like ionizing radiation by reducing free radicals and active oxygen species, according to several cellular and preclinical investigations (121).

Curcumin used with chemotherapy medications like docetaxel, 5-fluorouracil, doxorubicin, and cisplatin improves the synergistic effect by altering numerous signaling pathways, inhibiting tumors including prostate, hepatic, gastric, Hodgkin lymphoma, bladder, and colorectal cancers (122).

Curcumin is thought to have anti-cancer properties by interfering with several cellular processes and activating or inhibiting the production of certain cytokines, enzymes, and growth factors. Curcumin's anti-cancer potential, however, has been limited, owing to its low water solubility. Curcumin compounds with improved efficacy and/or water solubility or stability have resulted through chemical modification of these moieties (107).

### Black Cumin

*Nigella sativa* (*N. sativa*) is a tiny shrub with annual flowers that belongs to the Ranunculaceae family. It has white, pink, yellow, and purplish exquisite flowers with 5 to 10 petals (123). When the fruit is ripped open, it reveals a great number of black seeds known as black cumin in English, and Habbat el Baraka or Habbah Sawda in Arabic (124). Syria, Lebanon, Pakistan, India, and Afghanistan are among the Middle Eastern and Western Asian countries where the *N. sativa* plant is widely farmed. *N. sativa* are used as a spice in Indian and extensively in Middle Eastern cuisines due to its pungent bitter taste and aroma. The seeds contain many vitamins and minerals in addition to important active compounds including thymoquinone, thymohydroquinone and dithymoquinone (nigellone) (24).

**Figure 6:**
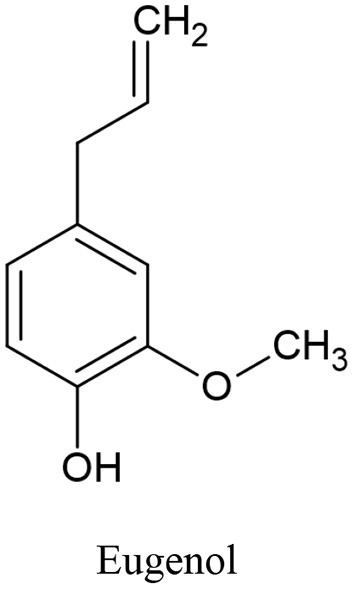
Eugenol chemical structure.

The pharmacological properties of *N. sativa* are mainly due to its quinine constituents, primarily thymoquinone (Figure 6) because it is the most abundant monoterpene (24).

Among several therapeutic plants, *N. Sativa* has long been regarded as one of the most valued nutrient-rich herbs in history, and various published scientific studies are currently ongoing to confirm the traditional applications of this species' small seed (72).

Because of its low toxicity and numerous mechanisms of action, *N. Sativa* can be a useful tool for health improvement (125). Recent research suggests that *N. Sativa* oil and extracts contain anti-inflammatory and antimicrobial characteristics, as well as bronchodilator, hypoglycemic, immune booster, anticancer and antioxidant properties (126–130). Once the antitumor characteristics of the *N. Sativa* seed and extracts were established, the researchers investigated the antitumor properties of its major active components, such as thymoquinone and dithymoquinone (131). Black cumin's antitumor mechanism of action is as follows:

Thymoquinone (TQ) antioxidant and cytotoxic effect has been studied *in vitro* and *in vivo* utilizing a variety of animal models and tumor cell lines.

One of the first publications pointing to *N. sativa*'s possible anti-cancer characteristics, An aqueous extract of *N. Sativa* seeds were found to have considerable cytotoxic effects on tumor cell lines (HepG2, MOLT4, and LL/ 2), but not on healthy, non-cancerous umbilical cord endothelial cells (132).

Both aqueous and ethanolic extracts of *N. Sativa* seeds were also found to exhibit significant cytotoxic effects on MCF-7 cells in the presence and lack of H_2_O_2_, apart from their anti-proliferative properties (133).

A crude methanolic extract of *N. Sativa* also induced around 50% cytotoxicity in Sarcoma180 cells (S-180 cells), Dalton's lymphoma ascites, and Ehrlich ascites carcinoma, in an *in vitro* cytotoxic study (134).

Another *in vivo* study found that 6-month oral administration of *N. Sativa* seeds protected rats from methylnitrosourea-induced oxidative stress and colon carcinogenesis due to lower production of malondialdehyde (MDA), a lipid peroxidation biomarker, and nitric oxide (NO) biomarker (135).

A few researches has investigated the possibility of *N. Sativa* having an anti-mutagenic effect against the directly acting mutagen N-methyl-N0 -nitro-N-nitrosoguanidine (MNNG).

Due to dramatically reduced chromosomal abnormalities in primary rat hepatocytes, an ethanolic extract of *N. Sativa* exerted an inhibitory effect against MNNG mutagenicity. MNNG's anti-mutagenic actions were assigned to the stimulation of detoxifying enzymes that break down MNNG, chemical contact with or uptake of MNNG (or its electrophilic degradation products), increased DNA replication fidelity and enhanced DNA repair (136).

Several studies examined the impact of N. Sativa oil on the fibrinolytic capability of HT1080 human fibrosarcoma cell lines, which is a marker of malignant tumors.

In cell cultures, *N. Sativa* oil produced a dose-dependent downregulation of major fibrinolytic products such as urokinase-type plasminogen activator (u-PA), tissue-type plasminogen activator (tPA), and plasminogen activator inhibitor type 1. The capacity of N. Sativa to prevent local tumor invasion and metastasis is highlighted in this study (137).

In many studies, several research groups have postulated that increasing NK cytotoxic activity against cancer cells is a mechanism underlying *N. sativa*'s anti-cancer properties (138, 139).

The ability of *N. Sativa* to alter the activity of key enzymes has been primarily related to the key mechanisms underlying the reported anti-cancer properties of the plant (140, 141).

The inducible nitric oxide synthase (iNOS) pathway is one mechanism that has been linked to tumorigenesis. NO is an endogenous radical that is synthesized by iNOS or another NOS isoforms throughout physiological events such as inflammation and has been linked to tumor growth. In a recent study, they investigated how an ethanolic extract of N. Sativa would modify the iNOS pathway in rats with hepatocarcinogenesis induced by diethylnitrosamine (DENA). The serum levels of alpha-fetoprotein (AFP), NO, IL-6, and TNF-α factors whose production was considerably bolstered after treatment with DENA, were dramatically reduced after oral administration of N. Sativa ethanolic extract (142).

A study published recently found that a methanolic extract of *N. Sativa* seeds caused apoptosis in MCF7 cells in a dose- and time-dependent manner. In MCF7 cells, the methanolic extract of N. Sativa resulted in a significant increase in the expression of apoptotic factors such as caspase-3, caspase-8, caspase-9, and the p53 tumor protein, implying that *N. sativa*'s anti-cancer activity is mediated through the p53 and caspase signaling pathways (143).

Thymoquinone, the active phytochemical of *Nigella sativa*, exhibited an anticancer effect toward different cancer cells. It has suppressed the expression of janus Kinase 2 (Jak2) and STAT3, as well as upregulated the ROS level, and promoted apoptosis in human melanoma cells (144). Guler et al. reported the molecular anticancer activity of TQ in glioma cells. It has mediated apoptosis via inhibiting pSTAT3, hindering matrix metalloproteinases (MMP) and GSH levels, increasing iROS generation (145). Another study revealed the cytotoxic effect of TQ in Neuro-2a cells. The caspase-3 induction, KIAP protein reduction, and uprising of BAX/Bcl2 ratio have been observed upon the treatment with TQ (146, 147). Several studies demonstrated the antitumor mechanisms of action of thymoquinone, including its effect on the main cancer biomarkers and cell growth (148, 149). Hence, TQ can suppress NF-Kb, IL-8, PI3K/AKT, and MAPK as well as prevent cell migration by reducing the expression of N-cadherin gene (149–151).

### Clove

Cloves, *Syzygium aromaticum* L, dried buds, have long been used as a spice and in traditional Chinese and Indian medicine. Cloves include a diverse variety of bioactive components. Sesquiterpenes, volatile oil (eugenol), caryophyllene, tannins, and gum are among the major chemical constituents of cloves (152, 153).

**Figure 7:**
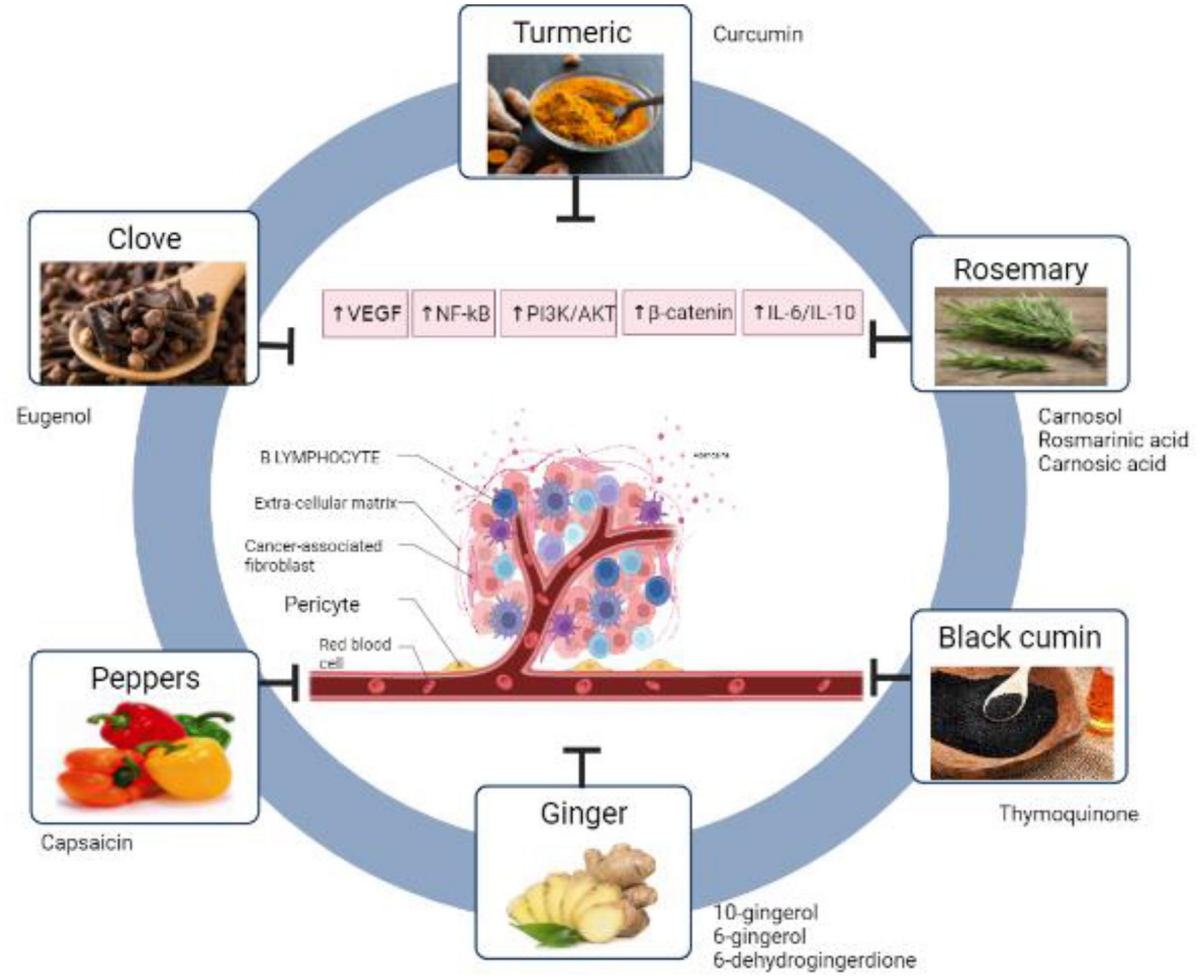
The main Mediterranean diet spices and their phytochemicals.

Clove oil is an effective antibacterial, analgesic, expectorant, antioxidant, and antispasmodic. Eugenol (Figure 7) is one of clove oil components that is responsible for its characteristic odor, is a colorless to pale yellow oily liquid and has been found in a few anticancer formulations (154).

Clove's antitumor mechanism of action as follows:

The capability to inhibit oxidative stress has been defined as a protective effect against cancer formation (carcinogenesis or tumorigenesis); however, whenever cancer has formed, the antioxidant effect can contribute to cancer's development, whereas the pro-oxidant effect can evoke cancer cell death through several signaling pathways (155, 156).

Notably, eugenol has been identified as an agent having a dual effect, antioxidant, and pro-oxidant, with beneficial effects in both cancer prevention and treatment (157–159).

With eugenol antioxidant activity, as assessed by diverse models, It has a strong 2,2-diphenyl-1-picrylhydrazyl (DPPH) free radical-scavenging ability when it reacts with DPPH (160–162).

Eugenol also exhibits ferric ion (Fe3+) reducing ability and electron donor properties, allowing it to neutralize free radicals by producing stable products (162).

Furthermore, in many studies eugenol has been shown to reduce microsomal lipid peroxidation as well as iron and OH radical-induced lipid peroxidation in rat liver mitochondria. The production of thiobarbituric acid-reactive compounds was used to evaluate the antioxidant effect (160, 161).

Some inflammatory markers, such as inducible iNOS and COX-2 expression, as well as the levels of pro-inflammatory cytokines IL-6, TNF-α, and prostaglandin E2 (PGE2), were reduced in dimethylbenz[a]anthracen (DMBA)-exposed animals after treatment with eugenol, showing its anti-carcinogenic effect. Furthermore, in mouse skin with otetradecanoylphorbol-13-acetate-induced inflammation, eugenol was observed to decrease the activation of NF-B (157, 163).

According to certain research, eugenol can induce cytotoxicity at concentrations in the μM range. In the μM range, eugenol suppresses melanoma cell proliferation by causing cell cycle arrest in the S phase, followed by cell apoptosis (164).

In one study, HL-60 (human promyelocytic leukemia), HepG2 (human hepatocellular carcinoma), U-937 (human histiocytic lymphoma), 3LL (Lewis mouse lung carcinoma), and SNU-C5 (human colon carcinoma) lines are also inhibited by eugenol in the μM range. Also, DNA fragmentation, loss of mitochondrial transmembrane potential, Bax translocation, Bcl-2 reduction, cytochrome c release, and caspase-9 and−3 activation are all observed in cells treated with eugenol in the μM range, implying that eugenol causes cell apoptosis (165).

In another study, Eugenol in the μM range produced cytotoxicity and caused a rise in the G2/M phase in LNCaP (androgen-responsive human prostate cancer) and PC-3 (androgen-independent human prostate carcinoma) cell lines (166) (Figure 1). Demonstrate the six spices that have mentioned in this review with their main phytochemicals (Table 2). Summarize the anticancer activity of the main Mediterranean diet spices and their mechanisms of action.

**Table 2:** Anticancer activity of the main Mediterranean diet spices and their mechanisms of action.

**Type of spices**	**Active ingredients**	**Model of experiment**		**Anticancer mechanism of action**	**References**
Ginger	10-gingerol	Human colon cancer cells (HCT-116)	*In vitro*	Reduced MAPK Increased DNA accumulation in the sub-G1 phase	(48)
	6-gingerol	Human keratinocyte cell line (HaCaT)	*In vitro*	Suppressed cell growth by reducing MAPK and AP-1 signaling pathways	(49)
		Mouse skin cells (ICR mice)	*In vivo*	Inhibited NF-kB, p38, and COX-2 expression	(50)
		Oral cancer cells	*In vitro*	Induced cell apoptosis and cell cycle G2/M phase arrest Activated AMPK and suppressed AKT/mTOR signaling pathway	(51)
		Gastric cancer cells (HGC-27 and MGC-803)	*In vitro*	Inhibited cell proliferation, migration and invasion via modulating of PI3/AKT signaling pathway	(52)
		Oral and cervical carcinoma cells (SCC4, KB and HeLa)	*In vitro*	Enhanced apoptosis and cell cycle arrest	(53)
		Renal carcinoma cells (ACHN, 786-O, and 769-P)	*In vitro* *In vivo*	Induced cell cycle arrest via modulation of AKT-GSK 3β-cyclin D1 pathway	(54)
		Osteosarcoma cells	*In vitro*	Suppressed AMPK signaling	(55)
		Human pancreatic cells (PANC-1)	*In vitro*	Downregulation of the ERK/NF- κ B/Snail signal transduction pathway	(56)
		Lung cancer cells (A549)	*In vitro* *In vivo*	Inhibited cell growth via decreasing of USP14 expression	(58)
		Rat colonic adenocarcinoma	*In vivo* *In vitro*	Inhibited cell proliferation and angiogenic potential of endothelial cell tubule formation	(60)
		Renal carcinoma cells (786-O and ACHN) BALB/C nude mice	*In vitro* *In vivo*	Suppressed cell migration through downregulation of YAP level	(61)
	6-dehydrogingerdione	Human breast cancer cells (MDA-MB-231 and MCF-7)	*In vitro*	Induced cell apoptosis through oxygen species/c-Jun N-terminal kinase pathway	(57)
Peppers	Capsaicin	Human colorectal cells (HCT-116, SW480, and LoVo)	*In vitro*	Enhanced cell apoptosis by suppression transcriptional activity of β-catenin	(65)
		Human breast cancer cells (MDA-MB-231 and MCF-7)	*In vitro*	Suppressed cell proliferation and induced apoptosis by downregulation of FBI-mediated NF-kB pathway	(68)
		Human multiple myeloma cell lines (U266 and MM.1S) Male athymic nu/nu mice	*In vitro* *In vivo*	Inhibited the interleukin-6-induced STAT3 activation Suppressed tumor growth in mice	(70, 71)
		Non-small cell lung carcinoma cells (A549)	*In vitro*	Reduced cells angiogenesis by downregulation VEGF expression	(70, 71)
		Transgenic adenocarcinoma in mouse prostate model	*In vivo*	Reduced tumor growth and metastasis	(72)
Rosemary	Rosmarinic acid	Prostate cancer cell lines (PC-3 and DU145)	*In vitro*	Induced cell apoptosis through inhibition of HDAC2 expression	(90)
	Carnosol	BALB/C WEHI-164 fibrosarcoma model	*In vivo*	Inhibited tumor growth Decreased IL-4 and IL-10 Increased IFN production	(92)
Turmeric	Curcumin	Human epidermal keratinocytes	*In vitro*	Activated apoptosis by suppressing AP1 transcription dependent and Bcl-xL level	(109)
		Gastric and colon cancer cells (KATO-III and HCT-116)	*In vitro*	Induced apoptosis via upregulation of capase-3, PARP, and caspase-8 Reduced Bcl-xL level	(106, 107, 110)
		Hepatocellular carcinoma (H22HCC) Nude male mice	*In vitro* *In vivo*	Inhibited cell proliferation and induced apoptosis by decreasing VEGF expression and PI3K/AKT signaling	(112)
Black cumin	*N. sativa* extracts	Breast cancer cells (MCF-7)	*In vitro*	Reduced cells proliferation and enhanced apoptosis	(133)
		Sarcomal180 cells, Dalton's lymphoma ascites, Ehrlich ascites carcinoma	*In vitro* *In vivo*	Induced around 50% cytotoxicity Reduced tumor growth	(134)
		Hepatocellular carcinoma rats model	*In vivo*	Reduction of tumor growth via suppression of iNOS pathway and decreasing TNF-α and IL-6 levels	(142)
		Breast cancer cells (MCF-7)	*In vitro*	Induced apoptosis via increasing caspase-3, caspase-8, caspase-9, and p53 expression	(143)
	*N. sativa* oil	Human fibrosarcoma cell line (HT1080)	*In vitro*	Inhibited local tumor invasion and metastasis by downregulation u-PA, tPA, and PAI-1	(137)
	Thymoquinone	Human melanoma cells (SK-MEL-28) Xenograft mouse model	*In vitro* *In vivo*	Induced apoptosis by decrease the expression of Bcl-2, Bcl-xL,D cyclines, STAT3, and survivin Suppressed tumor growth in xenograft mouse model	(144)
		C6 glioma cells rats model	*In vivo*	Mediated apoptosis via inhibiting pSTAT3, hindering MMP, GSH levels, and increasing iROS generation	(145)
		Mouse neuroblastoma cells (Neuro-2a)	*In vitro*	Inhibited cell growth through caspase-3 induction, KIAP protein reduction, and uprising of Bax/Bcl2 ratio	(146)
		Breast cancer cells	*In vitro*	Inhibited PI3K/AKT1 pathway	(151)
Clove	Eugenol	Skin tumor in male Swiss albino mice model	*In vivo*	Decreased the activation of NF-kB	(157, 163)
		Human cervical cancer cells (HeLa)	*In vitro*	Induced apoptosis via downregulation of Bcl-2, COX-2, and IL-1β	(157, 163)
		Human melanoma cells Female B6D2F1 mice bearing B16 melanomas	*In vitro* *In vivo*	Suppressed tumor growth through inhibition of E2F1 transcriptional activity Tumor size decreased almost 40% compared to the control group	(164)
		Human promyelocytic leukemia (HL-60)	*In vitro*	Induced cell apoptosis through upregulation of Bax, caspase-3, caspase-9, and cytochrome c Decreased Bcl-2, and ROS expression	(165)
		Human prostate cancer cells (PC-3and DU 145)	*In vitro*	Produced cytotoxicity and caused a rise in the G2/M phase	(166)

## Conclusion

The clue in this review suggested that spices could be part of your daily diet that may lower cancer risk and affect tumor manner of acting. This review only scratches the surface of the overall impact of spices because roughly speaking there are 180 spices widely being used for several purposes. The proof goes on those numerous processes, involving proliferation, apoptosis, angiogenesis, signaling pathways, transduction, cell cycle phases, and immunocompetence could be affected by one or more of the previously mentioned spices, which in turn is reflected on the tumor activity. The Mediterranean diet is rich source of numerous spices. Compared with other diets, it includes multiple spices instead of focusing on single one. The presence of a cocktail of spices in single diet increases the chance of possible synergistic effect that may enhance the anticancer effect of standard therapies. The most common spice in the Mediterranean diet is black pepper (*Piper nigrum* L). Apoptosis induction is the most common anticancer pathway activated by different spices in the Mediterranean diet. Ginger and black cumin have the highest anticancer activities by targeting multiple cancer hallmarks. Further studies are needed to design anticancer diets containing the correct combination of spices.

## Author Contributions

All authors listed have made a substantial, direct, and intellectual contribution to the work and approved it for publication.

## Funding

The authors are grateful to the Applied Science Private University, Amman, Jordan, for the full financial support granted to this research (Grant No. DRGS-2020-2021-4).

## Conflict of Interest

The authors declare that the research was conducted in the absence of any commercial or financial relationships that could be construed as a potential conflict of interest.

## Publisher's Note

All claims expressed in this article are solely those of the authors and do not necessarily represent those of their affiliated organizations, or those of the publisher, the editors and the reviewers. Any product that may be evaluated in this article, or claim that may be made by its manufacturer, is not guaranteed or endorsed by the publisher.

## References

[1] Zheng J Zhou Y Li Y Xu D-P Li S Li H-B. Spices for prevention and treatment of cancers. Nutrients. (2016) 8:495. 10.3390/nu8080495 27529277 PMC4997408

[2] Kaliora AC Kountouri AM. Chemopreventive Activity of Mediterranean Medicinal Plants. Cancer Prevention–From Mechanisms to Translational Benefits. Rijeka: InTech (2012). p. 261–84.

[3] Bower A Marquez S De Mejia EG. The health benefits of selected culinary herbs and spices found in the traditional Mediterranean diet. Crit Rev Food Sci Nutr. (2016) 56:2728–46. 10.1080/10408398.2013.805713 25749238

[4] Vasilopoulou E Georga K Joergensen MB Naska A Trichopoulou A. The antioxidant properties of Greek foods and the flavonoid content of the Mediterranean menu. Curr Med Chem Immunol Endocr Metabol Agents. (2005) 5:33–45. 10.2174/1568013053005508

[5] Visioli F Grande S Bogani P Galli C. The role of antioxidants in the Mediterranean diets: focus on cancer. Eur J Cancer Prev. (2004) 13:337–43. 10.1097/01.cej.0000137513.71845.f6 15554562

[6] Issaoui M Delgado AM Caruso G Micali M Barbera M Atrous H. Phenols, flavors, and the mediterranean diet. J AOAC Int. (2020) 103:915–24. 10.1093/jaocint/qsz018 33241345

[7] Scalbert A Manach C Morand C Rémésy C Jiménez L. Dietary polyphenols and the prevention of diseases. Crit Rev Food Sci Nutr. (2005) 45:287–306. 10.1080/1040869059096 16047496

[8] Viuda-Martos M Ruiz Navajas Y Sánchez Zapata E Fernández-López J Pérez-Álvarez JA. Antioxidant activity of essential oils of five spice plants widely used in a Mediterranean diet. Flavour Fragr J. (2010) 25:13–9. 10.1002/ffj.1951

[9] Guasch-Ferré M Merino J Sun Q Fitó M Salas-Salvadó J. Dietary polyphenols, Mediterranean diet, prediabetes, and type 2 diabetes: a narrative review of the evidence. Oxid Med Cell Longev. (2017) 2017:6723931. 10.1155/2017/6723931 28883903 PMC5572601

[10] Bhosale PB Ha SE Vetrivel P Kim HH Kim SM Kim GS. Functions of polyphenols and its anticancer properties in biomedical research: a narrative review. Transl Cancer Res. (2020) 9:7619–31. 10.21037/tcr-20-2359 35117361 PMC8798728

[11] Patra S Pradhan B Nayak R Behera C Das S Patra SK. Dietary polyphenols in chemoprevention and synergistic effect in cancer: clinical evidences and molecular mechanisms of action. Phytomedicine. (2021) 90:153554. 10.1016/j.phymed.2021.153554 34371479

[12] Aggarwal BB Kunnumakkara AB Harikumar KB Tharakan ST Sung B Anand P. Potential of spice-derived phytochemicals for cancer prevention. Planta Med. (2008) 74:1560–9. 10.1055/s-2008-1074578 18612945

[13] Bhagat N Chaturvedi A. Spices as an alternative therapy for cancer treatment. Syst Rev Pharm. (2016) 7:46–56. 10.5530/srp.2016.7.7

[14] Bhathal SK Kaur H Bains K Mahal AK. Assessing intake and consumption level of spices among urban and rural households of Ludhiana district of Punjab, India. Nutr J. (2020) 19:1–12. 10.1186/s12937-020-00639-4 33158443 PMC7648309

[15] Peter KV Babu KN. Introduction to herbs and spices: medicinal uses and sustainable production. In: Handbook of Herbs And Spices. Amsterdam: Elsevier (2012). p. 1–16.

[16] Tsui P-F Lin C-S Ho L-J Lai J-H. Spices and atherosclerosis. Nutrients. (2018) 10:1724. 10.3390/nu10111724 30423840 PMC6266658

[17] Cassileth B. Mediterranean diet. Oncology. (2009) 23:1315. Available online at: http://www.ncbi.nlm.nih.gov/pubmed/20120847 20120847

[18] White B. Ginger: an overview. Am Fam Physician. (2007) 75:1689–91. Available online at: http://www.ncbi.nlm.nih.gov/pubmed/17575660 17575660

[19] Serra-Majem L Sánchez-Villegas A Román-Viñas B Guasch-Ferré M Corella D La Vecchia C. Mediterranean diet. Molecular Aspect Med. (2019) 67:1–55. 10.1016/j.mam.2019.06.001 31254553

[20] Guazzi E Maccioni S Monti G Flamini G Cioni PL Morelli I. *Rosmarinus officinalis* L. in the gravine of Palagianello. J Essential Oil Res. (2001) 13:231–3. 10.1080/10412905.2001.9699678

[21] Nieto G Ros G Castillo J. Antioxidant and antimicrobial properties of rosemary (*Rosmarinus officinalis*. L): a review. Medicines. (2018) 5:98. 10.3390/medicines5030098 30181448 PMC6165352

[22] Guynot ME Marin S Setu L Sanchis V Ramos AJ. Screening for antifungal activity of some essential oils against common spoilage fungi of bakery products. Food Sci Technol Int. (2005) 11:25–32. 10.1177/1082013205050901

[23] Khan A Chen HC Tania M Zhang DZ. Anticancer activities of Nigella sativa (black cumin). Afr J Tradit Complement Altern Med. (2011) 8(Suppl. 5):226–32. 10.4314/ajtcam.v8i5S.10 22754079 PMC3252704

[24] Srinivasan K. Cumin (Cuminum cyminum) and black cumin (Nigella sativa) seeds: traditional uses, chemical constituents, and nutraceutical effects. Food Qual Saf. (2018) 2:1–16. 10.1093/fqsafe/fyx031

[25] Ismail N Ismail M Azmi NH Bakar MFA Yida Z Abdullah MA. Thymoquinone-rich fraction nanoemulsion (TQRFNE) decreases Aβ40 and Aβ42 levels by modulating APP processing, up-regulating IDE and LRP1, and down-regulating BACE1 and RAGE in response to high fat/cholesterol diet-induced rats. Biomed Pharmacother. (2017) 95:780–8. 10.1016/j.biopha.2017.08.074 28892789

[26] Dhas PHA Korikanthimath VS. Processing and quality of black pepper: a review. J Spices Aromatic Crops. (2003) 12:1–14. Available online at: https://www.updatepublishing.com/journal/index.php/josac/article/view/4740

[27] Ahmad N Fazal H Abbasi BH Farooq S Ali M Khan MA. Biological role of piper nigrum L. (Black pepper): a review. Asian Pac J Trop Biomed. (2012) 2:S1945–53. 10.1016/S2221-1691(12)60524-3

[28] Serpa Guerra AM Gómez Hoyos C Velásquez-Cock JA Velez Acosta L Ganan Rojo P Velasquez Giraldo A M. The nanotech potential of turmeric (Curcuma longa L.) in food technology: a review. Crit Rev Food Sci Nutr. (2020) 60:1842–54. 10.1080/10408398.2019.1604490 31017458

[29] Rao PV Gan SH. Cinnamon: A Multifaceted Medicinal Plant. Evidence-Based Complement Altern Med. London: Hindawi Publishing Corporation (2014). 10.1155/2014/642942 PMC4003790 24817901

[30] Gruenwald J Freder J Armbruester N. Cinnamon and health. Crit Rev Food Sci Nutr. (2010) 50:822–34. 10.1080/10408390902773052 20924865

[31] Heywood V Skoula M. The MEDUSA network: conservation and sustainable use of wild plants of the Mediterranean region. Perspect N Crops N Use. (1999) 148:151.

[32] Scognamiglio M D'abrosca B Pacifico S Isidori M Esposito A Fiorentino A. Mediterranean Wild Plants As Useful Sources of Potential Natural Food Additives. In: Emerging Trends in Dietary Components for Preventing and Combating Disease. Washington, DC: ACS Publications (2012). 209–235.

[33] Fiorentino A Ricci A D'abrosca B Pacifico S Golino A Letizia M. Potential food additives from Carex distachya roots: identification and in vitro antioxidant properties. J Agric Food Chem. (2008) 56:8218–25. 10.1021/jf801603s 18686967

[34] Bosisio E Giavarini F Dell'agli M Galli G Galli C. Analysis by high-performance liquid chromatography of teucrin A in beverages flavoured with an extract of Teucrium chamaedrys L. Food Addit Contam. (2004) 21:407–14. 10.1080/02652030410001670157 15204541

[35] Goulas V Gomez-Caravaca AM Exarchou V Gerothanassis IP Segura-Carretero A Gutiérrez AF. Exploring the antioxidant potential of Teucrium polium extracts by HPLC–SPE–NMR and on-line radical-scavenging activity detection. LWT-Food Sci Technol. (2012) 46:104–9. 10.1016/j.lwt.2011.10.019

[36] Kabudari A Mahalleh S. Study of antibacterial effects of Teucrium polium essential oil on Bacillus cereus in cultural laboratory and commercial soup. Carpathian Food Sci Technol. (2016) 8:176–83. Available online at: https://www.researchgate.net/publication/311431950_Study_of_antibacterial_effects_of_Teucrium_polium_essential_oil_on_Bacillus_cereus_in_cultural_laboratory_and_commercial_soup

[37] D'abrosca B Pacifico S Scognamiglio M D'angelo G Galasso S Monaco P. A new acylated flavone glycoside with antioxidant and radical scavenging activities from Teucrium polium leaves. Nat Prod Res. (2013) 27:356–63. 10.1080/14786419.2012.695367 22708583

[38] D'abrosca B Fiorentino A Ricci A Scognamiglio M Pacifico S Piccolella S. Structural characterization and radical scavenging activity of monomeric and dimeric cinnamoyl glucose esters from *Petrorhagia velutina* leaves. Phytochem Lett. (2010) 3:38–44. 10.1016/j.phytol.2009.11.001

[39] Takwa S Caleja C Barreira JC Soković M Achour L Barros L. Arbutus unedo L. and Ocimum basilicum L. as sources of natural preservatives for food industry: a case study using loaf bread. LWT. (2018) 88:47–55. 10.1016/j.lwt.2017.09.041

[40] Aleksic V Knezevic P. Antimicrobial and antioxidative activity of extracts and essential oils of Myrtus communis L. Microbiol Res. (2014) 169:240–54. 10.1016/j.micres.2013.10.003 24291016

[41] Martinez-Tome M Jimenez AM Ruggieri S Frega N Strabbioli R Murcia M. Antioxidant properties of Mediterranean spices compared with common food *Additives*. (2001) 64:412–9. 10.4315/0362-028X-64.9.1412 11563520

[42] De Lima RMT Dos Reis AC De Menezes APM Santos JVO Filho J Ferreira JRO. Protective and therapeutic potential of ginger (Zingiber officinale) extract and [6]-gingerol in cancer: a comprehensive review. Phytother Res. (2018) 32:1885–907. 10.1002/ptr.6134 30009484

[43] Zhang M Zhao R Wang D Wang L Zhang Q Wei S. Ginger (Zingiber officinale Rosc.) and its bioactive components are potential resources for health beneficial agents. Phytother Res. (2021) 35:711–42. 10.1002/ptr.6858 32954562

[44] Ali BH Blunden G Tanira MO Nemmar AJF Toxicology C. Some phytochemical, pharmacological and toxicological properties of ginger (Zingiber officinale Roscoe): a review of recent research. Food Chem Toxicol. (2008) 46:409–20. 10.1016/j.fct.2007.09.085 17950516

[45] Mohd Yusof YA. Gingerol and its role in chronic diseases. Adv Exp Med Biol. (2016) 929:177–207. 10.1007/978-3-319-41342-6_8 27771925

[46] Chrubasik S Pittler MH Roufogalis BD. Zingiberis rhizoma: a comprehensive review on the ginger effect and efficacy profiles. Phytomedicine. (2005) 12:684–701. 10.1016/j.phymed.2004.07.009 16194058

[47] De Lima Silva WC Conti R De Almeida LC Morais PAB Borges KB Júnior VL. Novel [6]-gingerol Triazole Derivatives and their Antiproliferative Potential against Tumor Cells. Curr Top Med Chem. (2020) 20:161–9. 10.2174/1568026620666191227125507 31880263

[48] Ryu MJ Chung HS. [10]-Gingerol induces mitochondrial apoptosis through activation of MAPK pathway in HCT116 human colon cancer cells. In Vitro Cell Dev Biol Anim. (2015) 51:92–101. 10.1007/s11626-014-9806-6 25148824

[49] Davies M Robinson M Smith E Huntley S Prime S Paterson I. Induction of an epithelial to mesenchymal transition in human immortal and malignant keratinocytes by TGF-beta1 involves MAPK, Smad and AP-1 signalling pathways. J Cell Biochem. (2005) 95:918–31. 10.1002/jcb.20458 15861394

[50] Kim SO Chun KS Kundu JK Surh YJ. Inhibitory effects of [6]-gingerol on PMA-induced COX-2 expression and activation of NF-kappaB and p38 MAPK in mouse skin. Biofactors. (2004) 21:27–31. 10.1002/biof.552210107 15630166

[51] Zhang H Kim E Yi J Hai H Kim H Park S. [6]-Gingerol Suppresses Oral Cancer Cell Growth by Inducing the Activation of AMPK and Suppressing the AKT/mTOR Signaling Pathway. In Vivo. (2021) 35:3193–201. 10.21873/invivo.12614 34697150 PMC8627739

[52] Luo Y Zha L Luo L Chen X Zhang Q Gao C. [6]-Gingerol enhances the cisplatin sensitivity of gastric cancer cells through inhibition of proliferation and invasion via PI3K/AKT signaling pathway. Phytother Res. (2019) 33:1353–62. 10.1002/ptr.6325 30811726

[53] Kapoor V Aggarwal S Das SN. 6-gingerol mediates its anti tumor activities in human oral and cervical cancer cell lines through apoptosis and cell cycle arrest. Phytother Res. (2016) 30:588–95. 10.1002/ptr.5561 26749462

[54] Xu S Zhang H Liu T Yang W Lv W He D. 6-Gingerol induces cell-cycle G1-phase arrest through AKT-GSK 3β-cyclin D1 pathway in renal-cell carcinoma. Cancer Chemother Pharmacol. (2020) 85:379–90. 10.1007/s00280-019-03999-9 31832810 PMC7015962

[55] Riganti C Mini E Nobili S. Multidrug resistance in cancer: pharmacological strategies from basic research to clinical issues. Front Oncol. (2015) 5:105. 10.3389/fonc.2015.00105 26029662 PMC4426707

[56] Kim SO Kim MR. [6]-Gingerol prevents disassembly of cell junctions and activities of MMPs in invasive human pancreas cancer cells through ERK/NF- κ B/Snail signal transduction pathway. Evid Based Complement Alternat Med. (2013) 2013:761852. 10.1155/2013/761852 24204396 PMC3800596

[57] Hsu YL Chen CY Hou MF Tsai EM Jong YJ Hung CH. 6-Dehydrogingerdione, an active constituent of dietary ginger, induces cell cycle arrest and apoptosis through reactive oxygen species/c-Jun N-terminal kinase pathways in human breast cancer cells. Mol Nutr Food Res. (2010) 54:1307–17. 10.1002/mnfr.200900125 20175081

[58] Tsai Y Xia C Sun Z. The inhibitory effect of 6-Gingerol on Ubiquitin-specific peptidase 14 enhances autophagy-dependent ferroptosis and anti-tumor in vivo and in vitro. Front Pharmacol. (2020) 11:598555. 10.3389/fphar.2020.598555 33281606 PMC7691590

[59] Kim EC Min JK Kim TY Lee SJ Yang HO Han S. [6]-Gingerol, a pungent ingredient of ginger, inhibits angiogenesis in vitro and in vivo. Biochem Biophys Res Commun. (2005) 335:300–8. 10.1016/j.bbrc.2005.07.076 16081047

[60] Brown AC Shah C Liu J Pham JT Zhang JG Jadus MR. Ginger's (Zingiber officinale Roscoe) inhibition of rat colonic adenocarcinoma cells proliferation and angiogenesis in vitro. Phytother Res. (2009) 23:640–5. 10.1002/ptr.2677 19117330

[61] Xu S Zhang H Liu T Wang Z Yang W Hou T. 6-Gingerol suppresses tumor cell metastasis by increasing YAP(ser127) phosphorylation in renal cell carcinoma. J Biochem Mol Toxicol. (2021) 35:e22609. 10.1002/jbt.22609 32926756

[62] Batiha GE-S Alqahtani A Ojo OA Shaheen HM Wasef L Elzeiny M. Biological properties, bioactive constituents, and pharmacokinetics of some *Capsicum* spp. and capsaicinoids. Int J Mol Sci. (2020) 21:5179. 10.3390/ijms21155179 32707790 PMC7432674

[63] Reyes-Escogido MDL Gonzalez-Mondragon EG Vazquez-Tzompantzi EJM. Chemical and pharmacological aspects of capsaicin. Molecules. (2011) 16:1253–70. 10.3390/molecules16021253 21278678 PMC6259610

[64] Pramanik KC Boreddy SR Srivastava SK. Role of mitochondrial electron transport chain complexes in capsaicin mediated oxidative stress leading to apoptosis in pancreatic cancer cells. PLoS ONE. (2011) 6:e20151. 10.1371/journal.pone.0020151 21647434 PMC3102063

[65] Lee SH Richardson RL Dashwood RH Baek SJ. Capsaicin represses transcriptional activity of β-catenin in human colorectal cancer cells. J Nutr Biochem. (2012) 23:646–55. 10.1016/j.jnutbio.2011.03.009 21764279 PMC3197951

[66] Lee SH Krisanapun C Baek SJ. NSAID-activated gene-1 as a molecular target for capsaicin-induced apoptosis through a novel molecular mechanism involving GSK3beta, C/EBPbeta and ATF3. Carcinogenesis. (2010) 31:719–28. 10.1093/carcin/bgq016 20110283 PMC2847092

[67] Cunha MR Tavares MT Fernandes TB Parise-Filho R. Peppers: a “Hot” natural source for antitumor compounds. Molecules. (2021) 26. 10.3390/molecules26061521 33802144 PMC8002096

[68] Chen M Xiao C Jiang W Yang W Qin Q Tan Q. Capsaicin inhibits proliferation and induces apoptosis in breast cancer by down-regulating FBI-1-mediated NF-κB pathway. Drug Des Devel Ther. (2021) 15:125–40. 10.2147/DDDT.S269901 33469265 PMC7811378

[69] Min JK Han KY Kim EC Kim YM Lee SW Kim OH. Capsaicin inhibits in vitro and in vivo angiogenesis. Cancer Res. (2004) 64:644–51. 10.1158/0008-5472.CAN-03-3250 14744780

[70] Bhutani M Pathak AK Nair AS Kunnumakkara AB Guha S Sethi G. Capsaicin is a novel blocker of constitutive and interleukin-6-inducible STAT3 activation. Clin Cancer Res. (2007) 13:3024–32. 10.1158/1078-0432.CCR-06-2575 17505005

[71] Chakraborty S Adhikary A Mazumdar M Mukherjee S Bhattacharjee P Guha D. Capsaicin-induced activation of p53-SMAR1 auto-regulatory loop down-regulates VEGF in non-small cell lung cancer to restrain angiogenesis. PLoS ONE. (2014) 9:e99743. 10.1371/journal.pone.0099743 24926985 PMC4057320

[72] Venier NA Yamamoto T Sugar LM Adomat H Fleshner NE Klotz LH. Capsaicin reduces the metastatic burden in the transgenic adenocarcinoma of the mouse prostate model. Prostate. (2015) 75:1300–11. 10.1002/pros.23013 26047020

[73] Baugher PJ Krishnamoorthy L Price JE Dharmawardhane SF. Rac1 and Rac3 isoform activation is involved in the invasive and metastatic phenotype of human breast cancer cells. Breast Cancer Res. (2005) 7:1–10. 10.1186/bcr1329 16280046 PMC1410764

[74] Al-Sereiti MR Abu-Amer KM Sen P. Pharmacology of rosemary (Rosmarinus officinalis Linn.) and its therapeutic potentials. Indian J Exp Biol. (1999)37:124–30. 10641130

[75] Jianu C Goleţ I Stoin D Cocan I Lukinich-Gruia AT. Antioxidant activity of *Pastinaca sativa* L. ssp. *sylvestris [Mill.]* rouy and camus essential oil. Molecules. (2020) 25:869. 10.3390/molecules25040869 32079080 PMC7070583

[76] Xiang Q Liu Q Xu L Qiao Y Wang Y Liu XJFS. Carnosic acid protects biomolecules from free radical-mediated oxidative damage *in vitro*. Food Sci Biotechnol. (2013) 22:1–8. 10.1007/s10068-013-0226-2

[77] Satoh T Kosaka K Itoh K Kobayashi A Yamamoto M Shimojo Y. Carnosic acid, a catechol-type electrophilic compound, protects neurons both in vitro and in vivo through activation of the Keap1/Nrf2 pathway via S-alkylation of targeted cysteines on Keap1. J Neurochem. (2008) 104:1116–31. 10.1111/j.1471-4159.2007.05039.x 17995931 PMC4566957

[78] Ibáñez C Valdés A García-Cañas V Simó C Celebier M Rocamora-Reverte L. Global Foodomics strategy to investigate the health benefits of dietary constituents. J Chromatogr A. (2012) 1248:139–153. 10.1016/j.chroma.2012.06.008 22727325

[79] Greenberg ER Baron JA Tosteson TD Freeman DH Jr Beck GJ Bond JH. A clinical trial of antioxidant vitamins to prevent colorectal adenoma. polyp prevention study group. N Engl J Med. (1994) 331:141–7. 10.1056/NEJM199407213310301 8008027

[80] Baron JA Cole BF Mott L Haile R Grau M Church TR. Neoplastic and antineoplastic effects of beta-carotene on colorectal adenoma recurrence: results of a randomized trial. J Natl Cancer Inst. (2003) 95:717–22. 10.1093/jnci/95.10.717 12759389

[81] Mooney LA Madsen AM Tang D Orjuela MA Tsai WY Garduno ER. Antioxidant vitamin supplementation reduces benzo(a)pyrene-DNA adducts and potential cancer risk in female smokers. Cancer Epidemiol Biomarkers Prev. (2005) 14:237–42. Available online at: 10.1158/1055-9965.237.14.1 15668500

[82] Yoshinaka R Shibata M-A Morimoto J Tanigawa N Otsuki Y. COX-2 Inhibitor Celecoxib Suppresses Tumor Growth and Lung Metastasis of a Murine Mammary Cancer. Anticancer Res. (2006) 26:4245. Available online at: http://www.ncbi.nlm.nih.gov/pubmed/17201140 17201140

[83] Kirsh VA Kreiger N Cotterchio M Sloan M Theis B. Nonsteroidal Antiinflammatory Drug Use and Breast Cancer Risk: Subgroup Findings. Am J Epidemiol. (2007) 166:709–16. 10.1093/aje/kwm216 17660454

[84] Scheckel KA Degner SC Romagnolo DF. Rosmarinic acid antagonizes activator protein-1–dependent activation of cyclooxygenase-2 expression in human cancer and nonmalignant cell lines. J Nutr. (2008) 138:2098–105. 10.3945/jn.108.090431 18936204 PMC3151436

[85] Lin C-Y Wu C-R Chang S-W Wang Y-J Wu J-J Tsai C-W. Induction of the pi class of glutathione S-transferase by carnosic acid in rat Clone 9 cells via the p38/Nrf2 pathway. Food and Function. (2015) 6:1936–43. 10.1039/C4FO01131G 25974399

[86] López-Jiménez A García-Caballero M Medina MÁ Quesada AR. Anti-angiogenic properties of carnosol and carnosic acid, two major dietary compounds from rosemary. Eur J Nutr. (2013) 52:85–95. 10.1007/s00394-011-0289-x 22173778

[87] Yu Y-M Lin C-H Chan H-C Tsai H-D. Carnosic acid reduces cytokine-induced adhesion molecules expression and monocyte adhesion to endothelial cells. Eur J Nutr. (2009) 48:101. 10.1007/s00394-008-0768-x 19142568

[88] Johnson JJ. Carnosol: A promising anti-cancer and anti-inflammatory agent. Cancer Lett. (2011) 305:1–7. 10.1016/j.canlet.2011.02.005 21382660 PMC3070765

[89] Musolino C Sant'antonio E Penna G Alonci A Russo S Granata A. Epigenetic therapy in myelodysplastic syndromes. Eur J Haematol. (2010) 84:463–73. 10.1111/j.1600-0609.2010.01433.x 20192987

[90] Jang Y-G Hwang K-A Choi K-C. Rosmarinic acid, a component of rosemary tea, induced the cell cycle arrest and apoptosis through modulation of HDAC2 expression in prostate cancer cell lines. Nutrients. (2018) 10. 10.3390/nu10111784 30453545 PMC6266655

[91] Gómez De Cedrón M Laparra JM Loria-Kohen V Molina S Moreno-Rubio J Montoya JJ. Tolerability and safety of a nutritional supplement with potential as adjuvant in colorectal cancer therapy: a randomized trial in healthy volunteers. Nutrients. (2019) 11:2001. 10.3390/nu11092001 31450563 PMC6769991

[92] Rahnama M Mahmoudi M Zamani Taghizadeh Rabe S Balali-Mood M Karimi G Tabasi N. Evaluation of anti-cancer and immunomodulatory effects of carnosol in a Balb/c WEHI-164 fibrosarcoma model. J Immunotoxicol. (2015) 12:231–8. 10.3109/1547691X.2014.934975 25027673

[93] Petiwala SM Berhe S Li G Puthenveetil AG Rahman O Nonn L. Rosemary (Rosmarinus officinalis) extract modulates CHOP/GADD153 to promote androgen receptor degradation and decreases xenograft tumor growth. PLoS ONE. (2014) 9:e89772. 10.1371/journal.pone.0089772 24598693 PMC3943728

[94] Petiwala SM Johnson JJ. Diterpenes from rosemary (Rosmarinus officinalis): Defining their potential for anti-cancer activity. Cancer Lett. (2015) 367:93–102. 10.1016/j.canlet.2015.07.005 26170168

[95] Valdés A García-Cañas V Artemenko KA Simó C Bergquist J Cifuentes A. Nano-liquid chromatography-orbitrap ms-based quantitative proteomics reveals differences between the mechanisms of action of carnosic acid and carnosol in colon cancer cells. Mol Cell Proteomics. (2017) 16:8–22. 10.1074/mcp.M116.061481 27834734 PMC5217784

[96] Huang MC Chen HY Huang HC Huang J Liang JT Shen TL. C2GnT-M is downregulated in colorectal cancer and its re-expression causes growth inhibition of colon cancer cells. Oncogene. (2006) 25:3267–76. 10.1038/sj.onc.1209350 16418723

[97] Allegra A Innao V Gerace D Bianco O Musolino C. The metabolomic signature of hematologic malignancies. Leuk Res. (2016) 49:22–35. 10.1016/j.leukres.2016.08.002 27526405

[98] Catalán Ú Barrubés L Valls RM Solà R Rubió L. In vitro metabolomic approaches to investigating the potential biological effects of phenolic compounds: an update. Genomics Proteomics Bioinformatics. (2017) 15:236–45. 10.1016/j.gpb.2016.12.007 28549934 PMC5582796

[99] Rodríguez-Antona C. Pharmacogenomics of paclitaxel. Pharmacogenomics. (2010) 11:621–3. 10.2217/pgs.10.32 20415548

[100] Tai J Cheung S Wu M Hasman D. Antiproliferation effect of Rosemary (Rosmarinus officinalis) on human ovarian cancer cells in vitro. Phytomedicine. (2012) 19:436–43. 10.1016/j.phymed.2011.12.012 22325591

[101] Kotronoulas A Pizarro N Serra A Robledo P Joglar J Rubió L. Dose-dependent metabolic disposition of hydroxytyrosol and formation of mercapturates in rats. Pharmacol Res. (2013) 77:47–56. 10.1016/j.phrs.2013.09.001 24044986

[102] González-Vallinas M Molina S Vicente G Sánchez-Martínez R Vargas T García-Risco MR. Modulation of estrogen and epidermal growth factor receptors by rosemary extract in breast cancer cells. Electrophoresis. (2014) 35:1719–27. 10.1002/elps.201400011 24615943

[103] Allegra A Tonacci A Pioggia G Musolino C Gangemi S. Anticancer Activity of Rosmarinus officinalis L.: Mechanisms of Action and Therapeutic Potentials. Nutrients. (2020) 12:1739. 10.3390/nu12061739 32532056 PMC7352773

[104] Niranjan A Prakash D. Chemical constituents and biological activities of turmeric (Curcuma longa l.)-a review. J Food Sci Technol. (2008) 45:109. Available online at: https://www.researchgate.net/profile/Abhishek-Niranjan/publication/283863862_Chemical_constituents_and_biological_activities_of_turmeric_Curcuma_longa_L_-A_review/links/5c1789a7299bf139c75e8c08/Chemical-constituents-and-biological-activities-of-turmeric-Curcuma-longa-L-A-review.pdf

[105] Soleimani V Sahebkar A Hosseinzadeh H. Turmeric (Curcuma longa) and its major constituent (curcumin) as nontoxic and safe substances. Phytother Res. (2018) 32:985–95. 10.1002/ptr.6054 29480523

[106] Purpura M Lowery RP Wilson JM Mannan H Münch G Razmovski-Naumovski V. Analysis of different innovative formulations of curcumin for improved relative oral bioavailability in human subjects. Eur J Nutr. (2018) 57:929–38. 10.1007/s00394-016-1376-9 28204880 PMC5861163

[107] Tomeh MA Hadianamrei R Zhao X. A Review of Curcumin and Its Derivatives as Anticancer Agents. Int J Mol Sci. (2019) 20:1033. 10.3390/ijms20051033 30818786 PMC6429287

[108] Tuorkey M. Curcumin a potent cancer preventive agent: mechanisms of cancer cell killing. Interv Med Appl Sci. (2014) 6:139–46. 10.1556/imas.6.2014.4.1 25598986 PMC4274352

[109] Balasubramanian S Eckert RL. Curcumin suppresses AP1 transcription factor-dependent differentiation and activates apoptosis in human epidermal keratinocytes. J Biol Chem. (2007) 282:6707–15. 10.1074/jbc.M606003200 17148446

[110] Moragoda L Jaszewski R Majumdar AP. Curcumin induced modulation of cell cycle and apoptosis in gastric and colon cancer cells. Anticancer Res. (2001) 21:873–8. 10.1016/S0016-5085(08)83313-6 11396178

[111] Gupta SC Patchva S Aggarwal BB. Therapeutic roles of curcumin: lessons learned from clinical trials. AAPS J. (2013) 15:195–218. 10.1208/s12248-012-9432-8 23143785 PMC3535097

[112] Pan Z Zhuang J Ji C Cai Z Liao W Huang Z. Curcumin inhibits hepatocellular carcinoma growth by targeting VEGF expression. Oncol Lett. (2018) 15:4821–6. 10.3892/ol.2018.7988 29552121 PMC5840714

[113] Pulido-Moran M Moreno-Fernandez J Ramirez-Tortosa C Ramirez-Tortosa M. Curcumin and health. Molecules. (2016) 21:264. 10.3390/molecules21030264 26927041 PMC6273481

[114] Mantovani A. Molecular pathways linking inflammation and cancer. Curr Mol Med. (2010) 10:369–73. 10.2174/156652410791316968 20455855

[115] Shanmugam MK Rane G Kanchi MM Arfuso F Chinnathambi A Zayed ME. The multifaceted role of curcumin in cancer prevention and treatment. Molecules. (2015) 20:2728–69. 10.3390/molecules20022728 25665066 PMC6272781

[116] Mohamed SIA Jantan I Haque MA. Naturally occurring immunomodulators with antitumor activity: An insight on their mechanisms of action. Int Immunopharmacol. (2017) 50:291–304. 10.1016/j.intimp.2017.07.010 28734166

[117] Catanzaro M Corsini E Rosini M Racchi M Lanni C. Immunomodulators inspired by nature: a review on curcumin and echinacea. Molecules. (2018) 23:2778. 10.3390/molecules23112778 30373170 PMC6278270

[118] Momtazi-Borojeni AA Haftcheshmeh SM Esmaeili S-A Johnston TP Abdollahi E Sahebkar A. Curcumin: A natural modulator of immune cells in systemic lupus erythematosus. Autoimmun Rev. (2018) 17:125–35. 10.1016/j.autrev.2017.11.016 29180127

[119] Kasi PD Tamilselvam R Skalicka-Wozniak K Nabavi SF Daglia M Bishayee A. Molecular targets of curcumin for cancer therapy: an updated review. Tumor Biol. (2016) 37:13017–28. 10.1007/s13277-016-5183-y 27468716

[120] Mansouri K Rasoulpoor S Daneshkhah A Abolfathi S Salari N Mohammadi M. Clinical effects of curcumin in enhancing cancer therapy: a systematic review. BMC Cancer. (2020) 20:791. 10.1186/s12885-020-07256-8 32838749 PMC7446227

[121] Shafaghati N Hedayati M Hosseinimehr SJ. Protective effects of curcumin against genotoxicity induced by 131-iodine in human cultured lymphocyte cells. Pharmacogn Mag. (2014) 10:106–10. 10.4103/0973-1296.131020 24914274 PMC4048555

[122] Tan BL Norhaizan ME. Curcumin combination chemotherapy: The implication and efficacy in cancer. Molecules. (2019) 24:2527. 10.3390/molecules24142527 31295906 PMC6680685

[123] Alkhalaf M. Antimicrobial and anti-cancer activity of nigella sativa oil-a review. Aust J Basic Appl Sci. (2013) 7:505–14.

[124] Ismail MYM Yaheya M. Therapeutic role of prophetic medicine Habbat El Baraka (*Nigella sativa L*.)-A review. World Appl Sci J. (2009) 7:1203–8.

[125] Yimer EM Tuem KB Karim A Ur-Rehman N Anwar F. *Nigella sativa* L. (Black Cumin): a promising natural remedy for wide range of illnesses. Evid Base Complement Alternat Med. (2019) 2019:1528635. 10.1155/2019/1528635 31214267 PMC6535880

[126] El-Dakhakhny M Mady NI Halim MA. *Nigella sativa* L. oil protects against induced hepatotoxicity and improves serum lipid profile in rats. Arzneimittelforschung. (2000) 50:832–6. 10.1055/s-0031-1300297 11050701

[127] Ikhsan M Hiedayati N Maeyama K Nurwidya F. Nigella sativa as an anti-inflammatory agent in asthma. BMC Res Notes. (2018) 11:744. 10.1186/s13104-018-3858-8 30340634 PMC6194640

[128] Almatroodi SA Almatroudi A Alsahli MA Khan AA Rahmani AH. Thymoquinone, an active compound of *Nigella sativa*: role in prevention and treatment of cancer. Curr Pharm Biotechnol. (2020) 21:1028–41. 10.2174/1389201021666200416092743 32297580

[129] Hossain MS Sharfaraz A Dutta A Ahsan A Masud MA Ahmed IA. A review of ethnobotany, phytochemistry, antimicrobial pharmacology and toxicology of Nigella sativa L. Biomed Pharmacother. (2021) 143:112182. 10.1016/j.biopha.2021.112182 34649338

[130] Kulyar MF Li R Mehmood K Waqas M Li K Li J. Potential influence of *Nagella sativa* (Black cumin) in reinforcing immune system: a hope to decelerate the COVID-19 pandemic. Phytomedicine. (2021) 85:153277. 10.1016/j.phymed.2020.153277 32773257 PMC7347483

[131] Ali BH Blunden G. Pharmacological and toxicological properties of *Nigella sativa*. Phytother Res. (2003) 17:299–305. 10.1002/ptr.1309 12722128

[132] Swamy SMK Tan BKH. Cytotoxic and immunopotentiating effects of ethanolic extract of *Nigella sativa* L. seeds. J Ethnopharmacol. (2000) 70:1–7. 10.1016/S0378-8741(98)00241-4 10720783

[133] Farah IO Begum RA. Effect of *Nigella sativa* (N. sativa L.) and oxidative stress on the survival pattern of MCF-7 breast cancer cells. Biomed Sci Instrum. (2003) 39:359–64. 12724920

[134] Salomi NJ Nair SC Jayawardhanan KK Varghese CD Panikkar KR. Antitumour principles from *Nigella sativa* seeds. Cancer Lett. (1992) 63:41–6. 10.1016/0304-3835(92)90087-C 1555206

[135] Mabrouk GM Moselhy SS Zohny SF Ali EM Helal TE Amin AA. Inhibition of methylnitrosourea (MNU) induced oxidative stress and carcinogenesis by orally administered bee honey and Nigella grains in Sprague Dawely rats. J Exp Clin Cancer Res. (2002) 21:341–6. 12385575

[136] Khader M Bresgen N Eckl PM. Antimutagenic effects of ethanolic extracts from selected Palestinian medicinal plants. J Ethnopharmacol. (2010) 127:319–24. 10.1016/j.jep.2009.11.001 19913082

[137] Awad EM. In vitro decreases of the fibrinolytic potential of cultured human fibrosarcoma cell line, HT1080, by *Nigella sativa* oil. Phytomedicine. (2005) 12:100–7. 10.1016/j.phymed.2003.09.003 15693715

[138] Abuharfeil NM Maraqa A Von Kleist S. Augmentation of natural killer cell activity *in vitro* against tumor cells by wild plants from Jordan. J Ethnopharmacol. (2000) 71:55–63. 10.1016/S0378-8741(99)00176-2 10904146

[139] Majdalawieh AF Hmaidan R Carr RI. Nigella sativa modulates splenocyte proliferation, Th1/Th2 cytokine profile, macrophage function and NK anti-tumor activity. J Ethnopharmacol. (2010) 131:268–75. 10.1016/j.jep.2010.06.030 20600757

[140] Chehl N Chipitsyna G Gong Q Yeo CJ Arafat HA. Anti-inflammatory effects of the *Nigella sativa* seed extract, thymoquinone, in pancreatic cancer cells. Hpb. (2009) 11:373–81. 10.1111/j.1477-2574.2009.00059.x 19768141 PMC2742606

[141] Abdel-Hamid NM Abdel-Ghany MI Nazmy MH Amgad SW. Can methanolic extract of Nigella sativa seed affect glyco-regulatory enzymes in experimental hepatocellular carcinoma? Environ Health Prev Med. (2013) 18:49–56. 10.1007/s12199-012-0292-8 22767221 PMC3541809

[142] Fathy M Nikaido T. In vivo modulation of iNOS pathway in hepatocellular carcinoma by *Nigella sativa*. Environ Health Prev Med. (2013) 18:377–85. 10.1007/s12199-013-0336-8 23609474 PMC3773091

[143] Alhazmi MI Hasan TN Shafi G Al-Assaf AH Alfawaz MA Alshatwi AA. Roles of p53 and caspases in induction of apoptosis in MCF- 7 breast cancer cells treated with a methanolic extract of *Nigella sativa* seeds. Asian Pac J Cancer Prev. (2014) 15:9655–60. 10.7314/APJCP.2014.15.22.9655 25520084

[144] Raut PK Lee HS Joo SH Chun K-S. Thymoquinone induces oxidative stress-mediated apoptosis through downregulation of Jak2/STAT3 signaling pathway in human melanoma cells. Food Chem Toxicol. (2021) 157:112604. 10.1016/j.fct.2021.112604 34627931

[145] Guler EM Sisman BH Kocyigit A Hatiboglu MA. Investigation of cellular effects of thymoquinone on glioma cell. Toxicol Rep. (2021) 8:162–70. 10.1016/j.toxrep.2020.12.026 33489775 PMC7806546

[146] Paramasivam A Sambantham S Shabnam J Raghunandhakumar S Anandan B Rajiv R. Anti-cancer effects of thymoquinone in mouse neuroblastoma (Neuro-2a) cells through caspase-3 activation with down-regulation of XIAP. Toxicol Lett. (2012) 213:151–9. 10.1016/j.toxlet.2012.06.011 22732633

[147] Alhmied F Alammar A Alsultan B Alshehri M Pottoo FH. Molecular mechanisms of thymoquinone as anticancer agent. Comb Chem High Throughput Screen. (2021) 24:1644–53. 10.2174/1386207323999201027225305 33115388

[148] Odeh LH Talib WH Basheti IA. Synergistic effect of thymoquinone and melatonin against breast cancer implanted in mice. J Cancer Res Ther. (2018) 14:324. 10.4103/0973-1482.235349 29970684

[149] Homayoonfal M Asemi Z Yousefi B. Potential anticancer properties and mechanisms of thymoquinone in osteosarcoma and bone metastasis. Cell Mol Biol Lett. (2022) 27:21. 10.1186/s11658-022-00320-0 35236304 PMC8903697

[150] Khan MA Tania M Wei C Mei Z Fu S Cheng J. Thymoquinone inhibits cancer metastasis by downregulating TWIST1 expression to reduce epithelial to mesenchymal transition. Oncotarget. (2015) 6:19580. 10.18632/oncotarget.3973 26023736 PMC4637306

[151] Zhou J Imani S Shasaltaneh MD Liu S Lu T Fu J. PIK3CA hotspot mutations p. H1047R and p H1047L sensitize breast cancer cells to thymoquinone treatment by regulating the PI3K/Akt1 pathway. Mol Biol Rep. (2022) 49:1799–816. 10.1007/s11033-021-06990-x 34816327

[152] Park IK Shin SC. Fumigant activity of plant essential oils and components from garlic (Allium sativum) and clove bud (Eugenia caryophyllata) oils against the Japanese termite (Reticulitermes speratus Kolbe). J Agric Food Chem. (2005) 53:4388–92. 10.1021/jf050393r 15913300

[153] Haro-González JN Castillo-Herrera GA Martínez-Velázquez M Espinosa-Andrews H. Clove essential oil (Syzygium aromaticum L Myrtaceae): extraction, chemical composition, food applications, and essential bioactivity for human health. Molecules. (2021) 26:6387. 10.3390/molecules26216387 34770801 PMC8588428

[154] Fujisawa S Murakami Y. Eugenol and its role in chronic diseases. Drug Discover Mother Nature. (2016) 929:45–66. 10.1007/978-3-319-41342-6_3 27771920

[155] Fang J Seki T Maeda H. Therapeutic strategies by modulating oxygen stress in cancer and inflammation. Adv Drug Deliv Rev. (2009) 61:290–302. 10.1016/j.addr.2009.02.005 19249331

[156] Gorrini C Harris IS Mak TW. Modulation of oxidative stress as an anticancer strategy. Nat Rev Drug Discov. (2013) 12:931–47. 10.1038/nrd4002 24287781

[157] Kaur G Athar M Alam MS. Eugenol precludes cutaneous chemical carcinogenesis in mouse by preventing oxidative stress and inflammation and by inducing apoptosis. Mol Carcinog. (2010) 49:290–301. 10.1002/mc.20601 20043298

[158] Pal D Banerjee S Mukherjee S Roy A Panda CK Das S. Eugenol restricts DMBA croton oil induced skin carcinogenesis in mice: downregulation of c-Myc and H-ras, and activation of p53 dependent apoptotic pathway. J Dermatol Sci. (2010) 59:31–9. 10.1016/j.jdermsci.2010.04.013 20537511

[159] Yan X Zhang G Bie F Lv Y Ma Y Ma M. Eugenol inhibits oxidative phosphorylation and fatty acid oxidation via downregulation of c-Myc/PGC-1β/ERRα signaling pathway in MCF10A-ras cells. Sci Rep. (2017) 7:12920. 10.1038/s41598-017-13505-x 29018241 PMC5634997

[160] Ito M Murakami K Yoshino M. Antioxidant action of eugenol compounds: role of metal ion in the inhibition of lipid peroxidation. Food Chem Toxicol. (2005) 43:461–6. 10.1016/j.fct.2004.11.019 15680683

[161] Nagababu E Rifkind JM Boindala S Nakka L. Assessment of antioxidant activity of eugenol in vitro and in vivo. Methods Mol Biol. (2010) 610:165–80. 10.1007/978-1-60327-029-8_10 20013178 PMC3202335

[162] Gülçin I. Antioxidant activity of eugenol: a structure-activity relationship study. J Med Food. (2011) 14:975–85. 10.1089/jmf.2010.0197 21554120

[163] Hussain A Brahmbhatt K Priyani A Ahmed M Rizvi TA Sharma C. Eugenol enhances the chemotherapeutic potential of gemcitabine and induces anticarcinogenic and anti-inflammatory activity in human cervical cancer cells. Cancer Biother Radiopharm. (2011) 26:519–27. 10.1089/cbr.2010.0925 21939359

[164] Ghosh R Nadiminty N Fitzpatrick JE Alworth WL Slaga TJ Kumar AP. Eugenol causes melanoma growth suppression through inhibition of E2F1 transcriptional activity. J Biol Chem. (2005) 280:5812–9. 10.1074/jbc.M411429200 15574415

[165] Yoo CB Han KT Cho KS Ha J Park HJ Nam JH. Eugenol isolated from the essential oil of Eugenia caryophyllata induces a reactive oxygen species-mediated apoptosis in HL-60 human promyelocytic leukemia cells. Cancer Lett. (2005) 225:41–52. 10.1016/j.canlet.2004.11.018 15922856

[166] Ghosh R Ganapathy M Alworth WL Chan DC Kumar AP. Combination of 2-methoxyestradiol (2-ME2) and eugenol for apoptosis induction synergistically in androgen independent prostate cancer cells. J Steroid Biochem Mol Biol. (2009) 113:25–35. 10.1016/j.jsbmb.2008.11.002 19084597

